# Inhibition of Adherence of *Mycobacterium avium* to Plumbing Surface Biofilms of *Methylobacterium* spp.

**DOI:** 10.3390/pathogens6030042

**Published:** 2017-09-14

**Authors:** Mari Carmen Muñoz Egea, Pan Ji, Amy Pruden, Joseph O. Falkinham

**Affiliations:** 1 Department of Clinical Microbiology, Fundación Jiménez Díaz Hospital, 28040 Madrid, Spain; mcme86@gmail.com; 2 Department of Civil and Environmental Engineering, Virginia Tech, Blacksburg, VA 24061, USA; jpan1@vt.edu (P.J.); apruden@vt.edu (A.P.); 3 Department of Biological Sciences, Virginia Tech, Blacksburg, VA 24061, USA

**Keywords:** *Mycobacterium avium*, *Methylobacterium*, adherence, biofilm formation

## Abstract

Both *Mycobacterium* spp. and *Methylobacterium* spp. are opportunistic premise plumbing pathogens that are found on pipe surfaces in households. However, examination of data published in prior microbiological surveys indicates that *Methylobacterium* spp. and *Mycobacterium* spp. tend not to coexist in the same household plumbing biofilms. That evidence led us to test the hypothesis that *Methylobacterium* spp. in biofilms could inhibit the adherence of *Mycobacterium avium*. Measurements of adherence of *M. avium* cells to stainless steel coupons using both culture and PCR-based methods showed that the presence of *Methylobacterium* spp. biofilms substantially reduced *M. avium* adherence and vice versa. That inhibition of *M. avium* adherence was not reduced by UV-irradiation, cyanide/azide exposure, or autoclaving of the *Methylobacterium* spp. biofilms. Further, there was no evidence of the production of anti-mycobacterial compounds by biofilm-grown *Methylobacterium* spp. cells. The results add to understanding of the role of microbial interactions in biofilms as a driving force in the proliferation or inhibition of opportunistic pathogens in premise plumbing, and provide a potential new avenue by which *M. avium* exposures may be reduced for at-risk individuals.

## 1. Introduction

Nontuberculous mycobacteria (NTM) are opportunistic human pathogens whose source of infection is the environment [1]. *Mycobacterium* species are found in drinking water distribution systems [2], hospitals [3], and household plumbing [4], and cause life-threatening pulmonary infections [5] that are difficult to treat [6]. The most common species associated with pulmonary infection in the United States is *Mycobacterium avium* [5,6].

The incidence of NTM disease in the United States and Canada is rising [7,8]. In Toronto (Canada), NTM disease incidence has risen from 1.5 to 9.0 per 100,000 over the period 1997–2003 [7]. Similarly, NTM disease is increasing in the United States, based on reports of NTM lung disease in hospitalized persons [8]. A major contributor to this increase is the fact that elderly, slender women, lacking any of the classic risk factors for NTM disease, have a greater tendency than the general population to develop NTM pulmonary disease [9,10,11]. It follows that as the population of the United States continues to age—25% of the US population will be over 60 years by 2025 [12]—the incidence of NTM pulmonary disease will continue to increase. Further, as NTM-infected patients are subject to reemergence of infection or reinfection by other environmental NTM [13], it is of value to identify measures to reduce NTM exposure.

Recently, it was shown that the DNA fingerprints of *M. avium* isolates recovered from both the biofilm and water from an *M. avium*-infected pulmonary patient’s shower were related to the patient’s *M. avium* isolate [14]. That study was followed by a report demonstrating the widespread presence and high numbers of *Mycobacterium* spp. and *M. avium* in showerhead biofilms across the United States [15]. Although not highlighted by the authors, examination of that data indicated a potentially important pattern; namely, the presence of a high proportion of pink-pigmented *Methylobacterium* spp. were associated with reduced numbers of *Mycobacterium* spp. and the presence of a high proportion of *Mycobacterium* spp. with a low proportion of *Methylobacterium* spp. [15]. Identical results were observed by cultivation of showerhead biofilms in households in Philadelphia, Pennsylvania [16].

Like *M. avium* and other NTM, *Methylobacterium* spp. are normal inhabitants of drinking water distribution systems [17,18,19,20,21] and plumbing in buildings, including hospitals [22,23]. Further, a substantial proportion of *Methylobacterium* spp. isolates are chlorine-resistant [24], form biofilms [25,26], and belong to the group of amoeba-resisting bacteria in drinking water [27]. Household plumbing is also a habitat, as *Methylobacterium* spp. have been shown to be abundant amongst DNA clones recovered from shower curtains [28].

In this study, it was hypothesized that the presence of the pink-pigmented *Methylobacterium* spp. will be associated with the absence of *Mycobacterium* spp., and that the presence of *Mycobacterium* spp. will be associated with the absence of *Methylobacterium* spp. Laboratory experiments were performed to identify the basis for the exclusion of *M. avium* by *Methylobacterium* spp. Exclusion of *M. avium* by *Methylobacterium* spp. could provide a new approach for limiting the exposure of at-risk individuals to *M. avium* and other NTM.

## 2. Results

### 2.1. Adherence Measurements

Stainless steel coupons, held in paddles in the CDC Biofilm Reactor, were exposed to suspensions of two different consortia of water-acclimated *Methylobacterium* spp. cells, or normal tap water, for 21 days at room temperature, to produce biofilms. The paddles and coupons with biofilms, and a control paddle and coupons lacking any biofilm (control), were washed twice in sterile tap water, and then placed in a suspension of water-acclimated *M. avium* cells (~10^5^ CFU/mL) in the CDC Biofilm Reactor. Immediately and after 1, 2, 3, and 6 h exposure, paddles and coupons were removed, coupons aseptically removed from the paddles, placed in 5 mL of sterile tap water, adherent cells suspended by vortexing, and the number of adherent *M. avium* cells measured as colony-forming units.

### 2.2. Methylobacterium Extorquens Adherence

Given that cells of *M. extorquens* and other *Methylobacterium* spp. aggregate spontaneously in broth media [21], an indicator of high hydrophobicity, it was hypothesized that, like *M. avium*, *Methylobacterium* spp. would readily adhere to surfaces and form biofilms. To test this, the adherence of water-acclimated cells of the *M. extorquens* strain to stainless steel coupons was measured in the presence and absence of Blacksburg tap water biofilms. The results (Table 1) demonstrated that *M. extorquens* cells readily adhered to the coupons, and that the presence of an existing tap water biofilm increased the extent of adherence. Adherence was apparently quite rapid, as a substantial number of *M. extorquens* cells adhered immediately (time 0) after exposing the *M. extorquens* cells to coupons with biofilms (Table 1). Approximately 5 min was required for removal of coupons and their transfer to centrifuge tubes, thus, the time 0 samples allowed for 5 min of adherence.

### 2.3. Methylobacterium spp. Inhibition of M. avium Adherence by Colony Count

**Figure 1:**
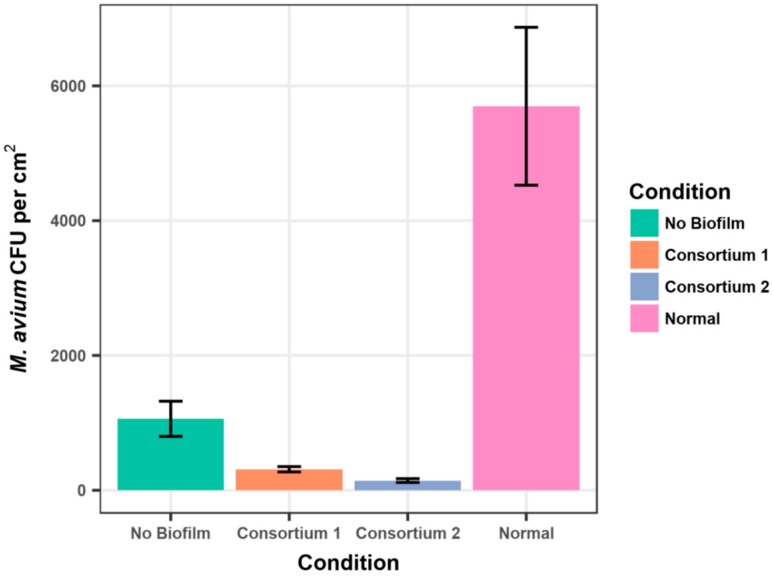
Adherence of *M. avium* strain A5 to stainless steel coupons after 6 h in the presence and absence of established normal tap water and *Methylobacterium* spp. biofilms measured by colony counts (±standard error).

The presence of established 21-day biofilms, composed of either Consortium 1 (*Methylobacterium* spp.) or Consortium 2 (*Methylobacterium* spp. and *Deinococcus grandis*), significantly reduced (ANOVA < 0.01) the adherence of *M. avium* strain A5 cells (Figure 1 and Supplementary Materials Table S1). The normal microbial biofilm produced by 21 days incubation of coupons in non-sterile Blacksburg tap water significantly (ANOVA *p* < 0.05) increased the adherence of the *M. avium* cells (Figure 1, Supplementary Materials Table S1), as noted for *M. extorquens* (Table 1). The results illustrated in Figure 1 reflect 6 h exposure to the *M. avium* suspension. Measurements of adherent *M. avium* cells at 0, 1, 2, and 3 h showed the same results, namely that *Methylobacterium* spp. consortium 1 and 2 inhibited the adherence of *M. avium* to the coupons (Supplementary Materials Table S1).

As these results came from short term adherence measurements (0 to 6 h), the conditions would have been unlikely to have had sufficient time to induce a viable, but nonculturable (VBNC) state in *M. avium*. Specifically, the loss of culturability of *M. avium* in biofilms requires at least one day [29]. To rule out the possibility that methylobacterial biofilms bound *M. avium* cells more tightly, and thereby, reduced suspension of adherent cells (thus reducing apparent adherence), coupons that had been treated to recover adherent mycobacteria were placed on M7H10 agar medium and incubated at 37 °C. Low numbers of *M. avium* colonies were observed (<10 colonies/coupon), even against the background of methylobacterial colonies that were low because of the relatively high incubation temperature. Such conditions would have also induced resuscitation of any VBNC mycobacterial cells over the course of incubation for 7 days on laboratory medium. We conclude that the reduced number of *M. avium* colonies in methylobacterial biofilms was not due to immediate induction of the VBNC state in mycobacterial cells.

As pink-pigmented yeast and cocci were recovered from patient household samples [16] and consortium 2 contained a strain of *Deinococcus grandis*, the adherence of *M. avium* strain A5 to stainless steel coupons was measured in the presence and absence of a yeast isolate 32-14 and the *D. grandis* strain JM-1-1. Neither the yeast, nor *D. grandis* biofilms reduced the adherence of *M. avium* strain A5 (Table 2). In fact, *D. grandis* biofilms appeared to have increased numbers of adherent *M. avium* cells (Table 2). In conclusion, the data support the hypothesis that the inhibition of *M. avium* adherence by Consortium 2 (Figure 1) was solely due to the *Methylobacterium* spp. cells.

### 2.4. Methylobacterium spp. Inhibition of M. avium Adherence by qPCR

One possible explanation for the reduced number of *M. avium* cells on *Methylobacterium* spp. biofilms (Figure 1, Supplementary Materials Table S1) could be that adherent *M. avium* cells lost their culturability and were unable to form colonies. To rule out that possibility, *M. avium* counts were measured by qPCR. Following suspension of biofilm-adherent cells, DNA was isolated, and *M. avium* gene copies enumerated by a sensitive and specific qPCR method [30]. The data, reported as *M. avium* gene copies/µL of isolated DNA, show that the number of gene copies on *Methylobacterium* spp. biofilms were below the limit of quantification (i.e., 3.95 × 10^6^ copies/cm^2^) immediately and after 1 and 3 h (Table 3). These results show that the reduction of *M. avium* counts on *Methylobacterium* spp. biofilms reflected by colony counts were not an artifact due to loss of culturability.

### 2.5. Inhibition of M. avium Adherence by Methylobacterium spp. Biofilms Does Not Require Methylobacterium spp. Viability

Three approaches were taken to measure whether viability of a *Methylobacterium* spp. biofilm was required for the inhibition of *M. avium* adherence: (1) poisoning with 10 mM cyanide and 10 mM azide (CN/AZ); (2) killing with ultraviolet-irradiation (UV); and (3) killing by autoclaving (Auto). The results demonstrated that *Methylobacterium* spp. biofilms whose survival was reduced to <100 CFU/cm^2^ by azide/cyanide-exposure, UV-irradiation, or autoclaving, still inhibited *M. avium* adherence (Table 4). The differences between the untreated control versus poisoned, versus UV-irradiated, or versus autoclaved *Methylobacterium* spp. biofilms on adherent *M. avium* colony counts were not significant (ANOVA > 0.05). In spite of the variation in the inhibition percentages, the data suggest that viability is not required for the inhibition of *M. avium* adherence to *Methylobacterium* spp. biofilms.

### 2.6. Methylobacterium spp. Biofilms Do Not Kill Adherent M. avium

To measure whether *Methylobacterium* spp. biofilms killed adherent *M. avium* cells, *M. avium* cells were exposed to a *Methylobacterium* spp. biofilm for 6 h to ensure adherence. Then, the coupons were removed from the *M. avium* suspension, washed, and placed in a CDC Biofilm Reactor containing sterile Blacksburg tap water. Immediately and daily (to 3 days), coupons were removed and the number of *M. avium* colony-forming units measured. Measurement of survival (CFU/cm^2^) of *M. avium* strain A5 cells adhering to *Methylobacterium* spp. biofilms showed no decrease over time; in fact, numbers increased (Table 5). That data rules out the possibility that cells of *M. avium* did adhere to *Methylobacterium* spp. biofilms, but were killed.

### 2.7. M. avium Strain A5 Biofilms Inhibit the Adherence of Methylobacterium spp. Cells to Stainless Steel

As only *Mycobacterium* spp. were present when *Methylobacterium* spp. were absent from biofilms of showerheads [15] or NTM patient household plumbing [16], the adherence of *Methylobacterium* spp. consortia to *M. avium* strain A5 biofilms was measured. The results show that 7-day *M. avium* biofilms reduced adherence of *Methylobacterium* spp. Consortium 1 cells, but only reduced the number of adherent cells of Consortium 2 after 3 h exposure to the *Methylobacterium* spp. suspension (Table 6).

## 3. Discussion

The data are consistent with the observations that *Methylobacterium* spp. and *M. avium* seldom are present in the same plumbing biofilm samples [15,16]. Further, the data support the hypothesis that the physical presence of either *Methylobacterium* spp. or *M. avium* in biofilms inhibits the adherence of the other species. Inspection of the Tables and Figures shows that there was substantial variation in the standard deviations of the average CFU/cm^2^ values. In part, this is due to the hydrophobicity-driven aggregation of the *M. avium* strain [31]. Colony counts of aggregates are subject to wide variation, as a sample may or may not contain aggregates with the same number of cells, and an aggregate can yield different colony counts depending upon the efficacy of spreading suspensions on agar media. For the experiments reported here, care was taken to ensure that suspensions were spread to dryness on 3-day agar media. Further, the data in each table present the results of the same experiment, using the same starting suspension of water-acclimated cells of *M. avium* to ensure that the different surfaces (e.g., present or absence of biofilm) could be made evident. At present, there are no alternatives to using aggregating mycobacterial strains as non-aggregating, hydrophilic derivatives are seldom isolated from patients and drinking water [1], and are thus unrepresentative. Likewise, aggregation appears to be a shared characteristic of *Methylobacterium* [21]. The use of detergents to produce uniform suspensions of cells is discouraged, as it alters surface hydrophobicity, and therefore, the natural behavior of these waterborne bacteria.

We conclude that the presence of an established *Methylobacterium* spp. biofilm substantially reduces the adherence and biofilm formation by *M. avium* cells. The inhibition in *M. avium* adherence did not require the viability of the *Methylobacterium* spp. cells, suggesting that the physical presence of *Methylobacterium* spp. cells is sufficient. It is possible that the first to attach may govern the further development of the biofilm microbial population. The presence of a normal, established microbial biofilm actually increased adherence of mycobacteria and methylobacteria. There was no demonstration of production of anti-mycobacterial activity by *Methylobacterium* spp. in biofilms, as the CFU/cm^2^ of *M. avium* cells adhering to *Methylobacterium* spp. biofilms did not decrease over time. That observation also rules out the possibility that nutrient competition is responsible, as *Methylobacterium* spp. are adapted to thrive at low nutrient concentrations [32].

Here, we describe a specific interaction between *M. avium* and *Methylobacterium* spp. in drinking water biofilms. Although these studies were focused on the interaction between *M. avium* and *Methylobacterium* spp., it is likely that other examples of inhibition of adherence will be found between other microorganisms. These observations contribute to an emerging understanding of drinking water biofilm microbiomes, and their importance in governing establishment and virulence of opportunistic pathogens [33]. The findings are particularly significant to those estimated 30,000 individuals in the United States with pulmonary mycobacterial disease [7,8], as they are innately susceptible to continued mycobacterial infections [13]. Specifically, the ecological interaction identified here in which *M. avium* adherence to biofilms is inhibited by *Methylobacterium* spp. could potentially be exploited as a strategy to limit adherence and biofilm formation by *M. avium* and possibly other *Mycobacterium* species, and reduce exposure of individuals to these opportunistic premise plumbing pathogens. This is in line with a “probiotic” framework recently suggested by Wang et al. [34]. Rather than suggest the “inoculation” of household plumbing with *Methylobacterium* spp. cells, we are investigating the possible inhibition of *M. avium* adherence by cellular fractions of *Methylobacterium* spp.

## 4. Materials and Methods 

### 4.1. Mycobacterium avium, Methylobacterium spp., Deinococcus grandis, and Yeast Isolates

*Mycobacterium avium* strain A5 is a plasmid-free clinical isolate [35]. The *Methylobacterium* spp. isolates were obtained from culture collections or pink-pigmented isolates recovered from showers and identified on the basis of 16S rRNA sequence (Table 7. The *D. grandis* isolate was included in Consortium 2, as it was pink-pigmented like *Methylobacterium* and recovered from a shower curtain. In addition, pink-pigmented yeast, strain P32-14, isolated from a shower curtain was included to rule out the possibility that it was responsible for the inhibition of *M. avium* adherence.

### 4.2. Preparation of M. avium Strain A5 and Methylobacterium spp. for Adherence Measurements

*M. avium* strain A5 was grown in 20 mL of Middlebrook 7H9 broth containing 0.5% (vol/vol) glycerol and 10% (vol/vol) oleic acid–albumin (M7H10) to mid log phase at 37 °C with aeration (60 rpm). *Methylobacterium* spp. strains were grown separately in 20 mL of Nutrient Broth (BD, Sparks, MD) to mid log phase at 30 °C with aeration. Following growth, cells of both *M. avium* and *Methylobacterium* spp. strains were collected by centrifugation (5000× *g* for 20 min), the supernatant medium discarded, and the cells were suspended in 20 mL of autoclaved Blacksburg tap water containing 0.05 mg humic acid/mL (Aldrich, St. Louis, MO, USA). Humic acid was added to provide a nutrient source that is common in drinking water. The suspensions were incubated at room temperature with aeration (60 rpm) for 7 days to acclimate to tap water. Two consortia of *Methylobacterium* spp. strains (Table 1) were prepared by mixing equal volumes of the water-acclimated suspensions.

### 4.3. CDC Reactor and Preparation for Adherence and Biofilm Measurements

CDC Biofilm Reactors (BioSurface Technologies Corp., Bozeman, MT, USA) were employed to measure adherence and biofilm formation on stainless steel coupons [36]. Stainless steel coupons were used as stainless steel pipes are present in household plumbing, relatively resistant to surface changes, and *M. avium* adherence is relatively high; though not as high as galvanized surfaces that are subject to surface changes over time [37]. Before use, the stainless steel coupons were thoroughly scrubbed and washed in detergent, rinsed, and dried, then soaked in 2 M HCl for 2 h, rinsed, dried, and placed into paddles of the CDC Biofilm Reactor.

### 4.4. Recovery and Enumeration of Adherent Cells by Colony Count

Paddles with coupons were removed from the CDC Biofilm Reactor and rinsed gently by immersion in sterile tap water twice. Coupons were aseptically removed from a paddle, and each coupon placed in 10 mL of sterile Butterfield buffer (per liter: 0.4 g KH_2_PO_4_, 1 gm peptone, and 20 mL Tween 80) contained in a 50 mL screw cap centrifuge tube. Each coupon was gently swirled to remove unattached cells, and the washed coupon drained and transferred to a second tube containing 10 mL fresh Butterfield buffer, and vortexed for 60 s. The undiluted and 10-, 100-, and 1000-fold diluted (Butterfield buffer) suspensions were spread (0.1 mL) in triplicate on either on M7H10 agar (*M. avium*) or R2A agar (*Methylobacterium* spp.), and incubated at either 37 °C (*M. avium*) or 30 °C (*Methylobacterium* spp.), and colonies with the appropriate pigmentation and morphology counted.

### 4.5. Recovery and Enumeration of Adherent Cells by qPCR

To complement the enumeration of adherent *M. avium* cells by colony count, qPCR was employed to measure numbers of adherent *M. avium* cells on *Methylobacterium* spp. biofilms. Paddles with coupons were removed from the CDC Biofilm Reactor and rinsed gently by immersion in sterile tap water twice. Coupons were aseptically removed from a paddle, and each coupon placed in a second tube containing 2 mL sterile tap water, and vortexed for 60 s. Following suspension of biofilm-adherent cells, an aliquot of 200 μL suspension was subject to DNA extraction using SPIN Kit (MP Biomedicals) according to the manufacturer’s instruction. Quantitative polymerase chain reaction (qPCR) was applied to measure the gene numbers of *M. avium* using previously established protocol [30]. The data was reported as *M. avium* gene copies/cm^2^.

### 4.6. Establishment of a Blacksburg Tap Water, Methylobacterium spp., Yeast, D. grandis, or M. avium Biofilms

The following 300 mL suspensions were added to separate sterile CDC Biofilm Reactor with paddles and coupons: (1) non-sterile Blacksburg tap water (normal biofilm flora); (2) a water-acclimated suspension of 10^5^ CFU *Methylobacterium* spp. Consortium 1 or 2/mL; (3) a water-acclimated suspension of 10^5^ CFU Philadelphia yeast isolate P32-14/mL; (4) a water-acclimated suspension of 10^5^ *D. grandis* strain JM-1-1/mL, or a water-acclimated suspension of 10^5^ *M. avium* strain A5 (for measurement of *M. extorquens* strain ATCC 43645 adherence to *M. avium* biofilms). The individual CDC reactors were incubated at room temperature for 21 days.

### 4.7. Measurement of M. avium Adherence to Stainless Steel in the Absence or Presence of A Methylobacterium spp. Biofilm

Following 21 days incubation to establish biofilms, paddles and coupons were removed, rinsed, and placed in a suspension containing 10^5^ CFU *M. avium* strain A5/mL. Immediately, and at 1, 3, and 6 h incubation at room temperature, a paddle with 3 coupons was removed and the number of adherent *M. avium* CFU measured, as described above.

### 4.8. Measurement of M. extorquens Adherence to Stainless Steel in the Absence or Presence of an M. avium Biofilm

For measurement of *M. extorquens* adherence, *M. avium* biofilm paddles and coupons were placed in a water-acclimated 10^5^ CFU *M. extorquens* ATCC strain 43,645/mL suspension. Results are expressed as *M. avium* or *M. extorquens* CFU/cm^2^ at each time point.

### 4.9. Is Methylobacterium spp. Viability Required for Inhibition of M. avium Adherence?

Three approaches were selected to reduce the viability of *Methylobacterium* spp. cells in biofilms: UV-irradiation, cyanide/azide-exposure, and autoclaving. Biofilms of Consortia 1 and 2 were established by incubation of the CDC reactor with stainless steel coupons. After 7 days incubation at room temperature, paddles and coupons were removed and separately washed in sterile tap water. Biofilms on coupons with consortium 1 or 2 biofilms were: (1) exposed to 1.5 mJ for 30 min and turned over for exposure of the opposite side for 30 min; (2) exposed to a 10 mM sodium azide (NaN_3_) and 10 mM potassium cyanide (KCN) solution for 60 min at room temperature; or (3) autoclaved in sterile water (15 min at 15 psi). After the three individual exposures and rinsing, the biofilms failed to yield viable *Methylobacterium* spp. CFU; survival <100/cm^2^. The exposed and unexposed (control) paddles with the coupons were washed by gentle immersion in sterile Blacksburg tap water, and placed in a CDC reactor containing a 10^5^ CFU *M. avium* strain A5/mL. *M. avium* adherence was measured immediately, and 2 and 3 h after placement of the coupons in the *M. avium* strain A5 suspension. Results are expressed as *M. avium* CFU/cm^2^ at 0, 2, and 3 h.

### 4.10. Measurement of Survival of Adherent M. avium by Methylobacterium spp. Biofilms

Based on the observation that a high frequency of anti-*Legionella pneumophila*-producing bacteria are present in biofilms [38], the survival of adherent *M. avium* cells in *Methylobacterium* spp. biofilms was measured. Biofilms of Consortia 1 and 2 were established by incubation of the CDC reactor with stainless steel coupons. After 7 days incubation at room temperature, paddles and coupons were removed and separately washed in sterile tap water. Paddles with *Methylobacterium* spp. biofilms were placed in a CDC Biofilm Reactor containing 10^5^ CFU *M. avium* strain A5/mL, and incubated for 6 h at room temperature to permit adherence of *M. avium* cells. The paddles were removed and gently rinsed in sterile Blacksburg tap water, and inserted into a sterilized CDC reactor containing only sterile Blacksburg tap water. Immediately and at daily intervals to 3 days, the number of adherent *M. avium* CFU/cm^2^ were measured as described above.

## Figures and Tables

**Table 1:** Adherence of *Methylobacterium extorquens* strain ATCC 43645 to stainless steel coupons in the presence and absence of an established tap water microbial biofilm ^1,2^.

Hours	*M. extorquens* CFU/cm^2^ No Biofilm	*M. extorquens* CFU/cm^2^ 21 d Tap Water Biofilm
0	19 ± 13	5200 ± 120 (<0.05)
1	190 ± 80	2300 ± 250 (<0.05)
2	680 ± 270	6900 ± 260 (<0.05)
3	1500 ± 450	3700 ± 240 (<0.05)
6	5700 ± 1200	7200 ± 160 (NS)

^1^ Average number of CFU/cm^2^ ± standard deviation adhering to each coupon type of triplicate measurements from two independent experiments. ^2^ (Statistical significance, ANOVA) compared to no biofilm. NS = not significant.

**Table 2:** *Mycobacterium avium* strain A5 Adherence to Biofilms of *Deinococcus grandis* strain JM-1-1 and Yeast strain 32-14 ^1^.

Hours	No Biofilm	*D. grandis* Biofilm	Yeast Biofilm
0	831 ± 390	1060 ± 240	1065 ± 300
1	482 ± 220	1570 ± 700	640 ± 640
2	890 ± 470	2810 ± 1,500	850 ± 900
3	2032 ± 700	4700 ± 1,700	1550 ± 350
6	1780 ± 450	3350 ± 1,200	1990 ± 210

^1^ Average number of CFU/cm^2^ ± standard deviation adhering to each coupon type of triplicate measurements from two independent experiments.

**Table 3:** Adherence of *M. avium* strain A5 to stainless steel coupons in the presence and absence of *Methylobacterium* spp. biofilms measured by qPCR ^1,2^.

Hours	*Methylobacterium* Consortium I
0	0 (non-detectable)
1	0 (non-detectable)
2	<3.95 × 10^6^ (3.95 × 10^6^ ± ×3.16 × 10^6^)
3	0 (non-detectable)
6	6.83 × 10^6^ ± 7.75 × 10^6^

^1^ Average number of *M. avium* gene copies/cm^2^ (±standard deviation) adhering to each coupon type of triplicate measurements from two independent experiments.

**Table 4:** Percent inhibition of 2 h *M. avium* adherence to untreated control, 10 mM azide/10 mM cyanide-exposed, 1.5 J UV-irradiated, and autoclaved biofilms of *Methylobacterium* spp. Consortia ^1,2^.

Treatment	Consortium 1	Consortium 2
Control	100%	100%
CN/AZ	99 ± 9	116 ± 12
UV	114 ± 87	113 ± 47
Autoclaved	146 ± 31	99 ± 54

^1^ Differences between Treatments not significant (ANOVA > 0.05). ^2^ Raw data in Supplementary Tables S2 and S3.

**Table 5:** Survival of *M. avium* strain A5 cells adhering to Consortium 1 and 2 biofilms ^1^.

Days	Consortium 1	Consortium 2
0 (Initial)	930 ± 670	150 ± 150
1	1300 ± 600	330 ± 53
2	1800 ± 900	570 ± 470
3	4600 ± 2100	610 ± 430

^1^ Average number of CFU/cm^2^ ± standard deviation adhering to each coupon type of triplicate measurements from two independent experiments.

**Table 6:** *M. avium* strain A5 biofilms inhibit the adherence of *Methylobacterium* spp. cells to stainless steel ^1^.

Hours	Consortium 1 No Biofilm	Consortium 1 *M. avium* Biofilm	Consortium 2 No Biofilm	Consortium 2 *M. avium* Biofilm
0	<20	<20	<20	<20
1	59 ± 24	39 ± 31	49 ± 22	103 ± 49
2	118 ± 78	59 ± 14	74 ± 16	89 ± 10
3	92 ± 65	30 ± 22	127 ± 41	64 ± 8

^1^ Average number of CFU/cm^2^ ± standard deviation adhering to each coupon type of triplicate measurements from two independent experiments.

**Table 7:** *Methylobacterium* spp. strains.

Consortium	Species	Strain	Source
1	*M. extorquens*	ATCC 43645	Soil, Japan
1	*M. aquaticum*	NCIMB 14006	Drinking Water, Seville
1	*M. adhaesivum*	NCIMB 14625	Drinking Water, Seville
1	*M. isbiliense*	NCIMB 14626	Drinking Water, Seville
1	*M. variable*	NCIMB 14628	Drinking Water, Seville
2	*M. hispanicum*	JM-5	Shower Curtain, USA
2	*M. hispanicum*	JM-8	Shower Curtain, USA

## Supplementary Materials

The following are available online at www.mdpi.com/ 2076-0817/6/3/42/s1, Table S1. Adherence of *M. avium* strain A5 to stainless steel coupons in the presence and absence of established normal microbial and *Methylobacterium* spp. biofilms measured by colony counts, Table S2. Effect of 10 mM azide and 10 mM cyanide exposure to *Methylobacterium* spp. biofilms on adherence of *M. avium* strain A5, Table S3. Effect of ultraviolet irradiation of *Methylobacterium* spp. biofilms on adherence of *M. avium* strain A5.
