# Supplementary material for: Inhibition of Adherence of Mycobacterium avium to Plumbing Surface Biofilms of Methylobacterium spp
Source: Pathogens. 2017 Sep 14;6(3):42. doi: 10.3390/pathogens6030042 (PMC5617999; doi:10.3390/pathogens6030042)
Supplement: Supplementary file 1 [file pathogens-06-00042-s001.pdf]

Supplementary Matter

***Methylobacterium* spp. Inhibition of *M. avium* Adherence**

Table S1. Adherence of *M. avium* strain A5 to stainless steel coupons in the presence and absence of established normal microbial and *Methylobacterium* spp. biofilms measured by colony counts[1, 2]

Hours	No Biofilm	Normal Flora	*Methylobacterium* Consortium 1	*Methylobacterium* Consortium 2
0	130 ± 11	690 ± 48 (< 0.05)	8.9 ± 1.8 (< 0.01)	5.5 ± 1.2 (< 0.01)
1	100 ± 5	1,600 ±132 (< 0.05)	64 ± 11 (NS)	66 ± 5.5 (NS)
2	400 ± 28	2,050 ± 52 (< 0.05)	45 ± 5.6 (< 0.001)	13 ± 1.6 (< 0.001)
3	430 ± 33	1,900 ± 68 (< 0.05)	76 ± 13 (< 0.05)	28 ± 3.2 (< 0.01)
6	1,100 ± 78	5,600 ± 342 (< 0.05)	220 ± 30 (< 0.05)	148 ± 8 (< 0.001)

[1]Average number of CFU/cm^2 ± standard deviation adhering to each coupon type of triplicate measurements from two independent experiments.

[2] (Statistical significance, ANOVA) compared to no biofilm

Table S2. Effect of 10 mM azide and 10 mM cyanide exposure to *Methylobacterium* spp. biofilms on adherence of *M. avium* strain A5[1]

Hours	Consortium 1 Live	Consortium 1 Poisoned	Consortium 2 Live	Consortium 2 Poisoned
0	< 200	< 200	< 200	< 200
2	7,900 ± 2,300	9,800 ± 2,200	9,800 ± 2,000	11,000 ± 5,000
3	15,000 ± 2,000	15,000 ± 1,000	20,000 ± 3,000	23,000 ± 2,000

[1]Average number of CFU/cm^2 ± standard deviation adhering to each coupon type of triplicate measurements from two independent experiments.

Table S3. Effect of ultraviolet irradiation of *Methylobacterium* spp. biofilms on adherence of *M. avium* strain A5 [1]

Hours	Consortium 1 Live	Consortium 1 UV-Irradiated	Consortium 2 Live	Consortium 2 UV-Irradiated
0	200 ± 180	190 ± 100	390 ± 80	340 ± 170
2	2,900 ± 1,700	3,600 ± 1,500	4,300 ± 2,200	5,100 ± 1,500
3	2,800 ± 1,100	3,500 ± 2,700	3,400 ± 2,200	5,300 ± 1,400

[1] Average number of CFU/cm^2 ± standard deviation adhering to each coupon type of triplicate measurements from two independent experiments
